# Characterization and Polydispersity of Volcanic Ash Nanoparticles in Synthetic Lung Fluid

**DOI:** 10.3390/toxics11070624

**Published:** 2023-07-19

**Authors:** Benedetto Schiavo, Ofelia Morton-Bermea, Diana Meza-Figueroa, Mónica Acosta-Elías, Belem González-Grijalva, Maria Aurora Armienta-Hernández, Claudio Inguaggiato, Daisy Valera-Fernández

**Affiliations:** 1 Instituto de Geofísica, Universidad Nacional Autónoma de México, Mexico City 04510, Mexico; 2 Departamento de Geología, Universidad de Sonora, Hermosillo 83000, Mexico; 3 Departamento de Investigación en Física, Universidad de Sonora, Hermosillo 83000, Mexico; 4 Departamento de Geología, Centro de Investigación Científica y de Educación Superior de Ensenada, Baja California (CICESE), Ensenada 22860, Mexico

**Keywords:** nanoparticles, volcanic ash, SEM, DLS, natural hazard, lung fluid, Popocatépetl

## Abstract

The inhalation of natural nanoparticles (NPs) emitted from volcanic activity may be a risk to human health. However, the literature rarely reports the fate and response of NPs once in contact with lung fluids. In this work, we studied the particle size distribution of ashfall from Popocatépetl volcano, Mexico. The collected ashes (n = 5) were analyzed with scanning electron microscopy (SEM) to obtain the elemental composition and morphology, and to determine the size of the ash particles using ParticleMetric software (PMS). The PMS reported most of the ash to have submicrometric size (<1 μm) and an average equivalent circle of 2.72 μm. Moreover, to our knowledge, this study investigated for the first time the behavior of ash NPs at different times (0 to 24 h) while in contact with in vitro lung fluid, Gamble Solution (GS) and Artificial Lysosomal Fluid (ALF) using dynamic light scattering (DLS). We found a large variability in the hydrodynamic diameter, with values less than 1 nm and greater than 5 μm. Furthermore, aggregation and disaggregation processes were recognized in GS and ALF, respectively. The results of this study increase the knowledge of the interaction between NPs and lung fluids, particularly within the alveolar macrophage region.

## 1. Introduction

Nanoparticles (NPs) play a fundamental role in the dynamics of Earth system and biogeochemical processes [1]. About 90% of aerosol NPs in the environment come from a natural source [2]. Humans, from prehistoric times to the present, has developed and manipulated several nanoscale materials in synthetic chemical processes [3]. Natural NPs contain a high degree of essential nutrients that can increase the productivity of microorganisms. Nanominerals may have affected the polymerization of the molecular building blocks and promoted the origin and evolution of bacterial cells. Additionally, nanominerals are involved in ocean fertilization, plankton bioproduction, carbon cycle, and climate change [4].

Natural NPs are characterized by very small size, particular optical properties, and large surface-to-volume ratios, showing specific physical and chemical attributes [5]. Particles present in the atmosphere with sizes ranging from 1 to 100 nm in diameter are usually classified as NPs [1]. According to Slezakova et al. [6] and Sonwani et al. [7], through atmospheric aggregation, NPs act as precursors to form larger particles that can influence climate on a global scale and are responsible for cooling effects and global warming. Compared with coarse particles with a residence time in the atmosphere of about one week [7], NPs have a much longer residence time due to the higher probability of resuspension [8].

The natural sources of atmospheric NPs could be biomass burning, sea spray, mineral dust, and volcanic eruption [8]. Dust mineral storms and volcanic activity are the principal sources of natural NPs in the atmosphere. Recent studies [9] have estimated the emission of NPs from mineral dust and volcanic eruptions to be 342,000 tons per year. After a single eruption, volcanoes can generate large amounts (tens of thousands of tons) of nanosized ash. During strong eruptions, the ash NPs reach the stratosphere and spread across the globe affecting the climate and ecosystem for years in different regions [10]. The impact of volcanic ash on the environment (water, soil, and air) is significant during recurrent eruptions and increases depending on the elemental composition of the released particles [11]. Once volcanic ash is injected into the atmosphere, the particles remain suspended for a period of time, settling on the land surface or water bodies such as lakes, rivers, and oceans through agglomeration mechanisms and dry and wet deposition. Faucher et al. [12] report that water is an important diffusion vector and reactivity medium for volcanic ash interactions.

Due to their ultrafine size, NPs are considered potential contaminants harmful to aquatic and terrestrial organisms, including human beings [11,13,14]. The toxicity of NPs also depends on their shape, origin, reactivity, surface chemistry, and charge. For their properties, NPs enter the human body via multiple exposure pathways, such as dermal, ingestion, and inhalation. Inhalation exposure is the primary pathway of NPs entering the body across the respiratory system, tending to deposit in the alveolar region [15]. The presence of NPs in the deep lung area causes oxidative stress and a chronic inflammatory response. Furthermore, Oberbek et al. [16] described how inhaled NPs can reach the bloodstream, pass through cell membranes, accumulate in vital organs, and cross the blood–brain barrier. NPs’ interaction with cells causes DNA and protein damage and affects basic cellular processes such as lysis, metabolism, and proliferation [17]. Several respiratory and cardiovascular diseases correlate with exposure to TiO_2_ and ZnO NPs [18].

As mentioned, the health risks associated with exposure to volcanic ash have been extensively documented [19,20]. Volcanic ash NPs are highly hazardous to human health, as they are able to penetrate deep into the respiratory system. The size and shape of ash particles are of critical importance in health. In the published literature [4,11,21,22,23], various authors have reported the transport, speciation, elemental composition, mineralogy, and pulmonary bioaccessibility of airborne volcanic ash. In particular, fractions smaller than 2 μm are associated with chronic diseases such as silicosis and pneumoconiosis [24]. However, the behavior of volcanic ash NPs’ interaction in contact with lung fluids is poorly studied. The main objectives of this work were (i) to determine the composition, morphology, and grain size distribution of volcanic ashfall through semi-quantitative analysis with scanning electron microscope–energy dispersive X-ray spectroscopy (SEM-EDS); (ii) to assess the behavior of volcanic ash NPs in contact with synthetic lung fluids (SLFs), Gamble Solution (GS) and Artificial Lysosomal Fluid (ALF); and (iii) to evaluate the polydispersity and hydrodynamic diameter of NPs in SLF over time (from 0 to 24 h) using dynamic light scattering (DLS). This study was conducted on several ashfall samples emitted from Popocatépetl volcano (Mexico) to reach the abovementioned objectives. Moreover, the nanometric size of the volcanic ash emitted by Popocatépetl and its impact on human health were evaluated for the first time.

## 2. General Background

### 2.1. Volcanic Ash Nanoparticles

In the literature, the study of NPs from volcanic emission and their toxicological effects have been reported, especially in the last two decades. Before this period, studies of particles <1 μm were rarely reported, except for some works concerning the determination and evolution of ultrafine volcanic ash emitted by Etna [25] and Mount St. Helens [26]. Several techniques are used to identify NPs (<100 nm) in volcanic ash and evaluate their toxic effect. Ermolin et al. [4], studying different Kamchatka volcanoes, describe a separation process of NPs using coiled-tube field-flow fractionation (CTFFF) performed by a rotation column, carrier fluid (generally Milli-Q water), and UV detector for system control. A complete scheme and basic principle of the planetary motion system can be found in Ermolin et al. [11] and Faucher et al. [12]. Subsequently, the separated ash fraction can be characterized by DLS after mixing it with deionized water and generating an NPs suspension.

The mobility, bioavailability, and toxicity of ash NPs are closely related to their content, chemical species, and mineralogy [10]. The physicochemical speciation and elemental concentration of NPs are analyzed using inductively coupled plasma (ICP)-AES (atomic emission spectroscopy) [11] and ICP-MS (mass spectrometry) [12]. Recently, an analytical technique used to analyze the concentration of metalloids in selected volcanic ash NPs has been single-particle (SP) ICP-MS, which can detect a single particle with a low detection limit [4]. The volcanic ash from Nevado del Ruiz (Colombia) was also studied using field-emission (FE) SEM and high-resolution transmission electron microscopy (HR-TEM) [23]. Trejos et al. [23] report a semi-quantitative analysis of volcanic ash deposited in the road dust of Manizales, identifying several mineralogical phases originating from volcanic emissions and NPs of magnetite and iron (Fe) and silicon (Si) amorphous phases, in a range between 10 and 200 nm.

The toxicity of volcanic ashfall particles has been evaluated mainly through correlations between exposure and respiratory effects [27,28], as well as in vivo and in vitro analysis [29]. Horwell and Baxter [15] report acute (asthma and bronchitis) and chronic (silicosis, pneumoconiosis, and pulmonary disease) health effects from prolonged ash inhalation in a population living close to the volcano area (Mt. St. Helens, USA, and Soufrière Hills, Montserrat). More recently, in vitro techniques have been developed to reproduce lung fluids (GS and ALF) and make them react with volcanic ash to evaluate bioaccessibility and deposition rate into the alveolar region [30]. Lähde et al. [21], studying ash NPs from Icelandic volcanoes, report that approximately 9% of the ash surface area is deposited into the deep lung, whereas the main fraction (fine particles) is deposited in the head airways region. On the other hand, the analysis of in vitro lung bioaccessibility and its standardization considering volcanic particles made it possible to determine the element hazard from inhaled ash [31].

### 2.2. Popocatépetl Eruptive History

**Figure 1:**
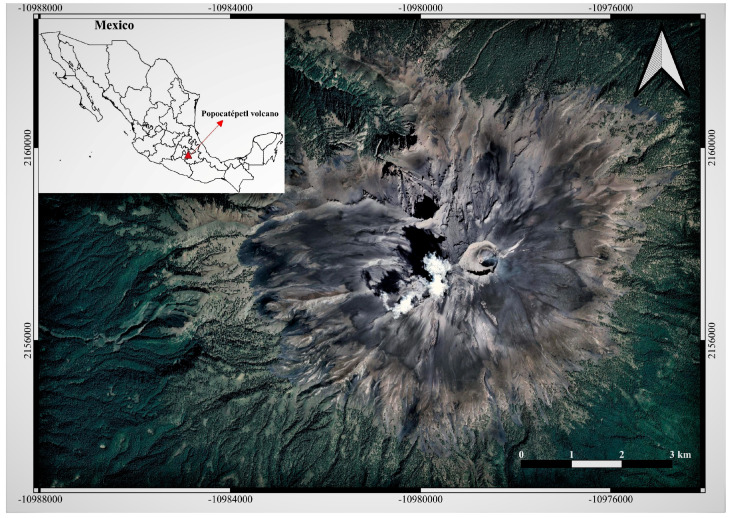
Location and satellite view of Popocatépetl volcano.

Popocatépetl volcano (Figure 1), one of the most active volcanoes in Mexico and Latin America, is located in the central sector of the Trans-Mexican Volcanic Belt (TMVB) and, along with two other inactive volcanoes, Tláloc and Iztaccíhuatl, forms the north–south-trending highland [32,33]. Popocatépetl, during the past 23,000 years, has produced several eruptions, including massive collapses due to Plinian events that took place on the order of 1000 and 3000 years ago [34], and lower-intensity eruptions recorded between AD 1500 and the end of 1800 [35]. Archaeological evidence, such as ceramic fragments buried by ashes, established that the most recent Plinian eruption occurred between AD 675 and 1095 [33]. Martin-Del Pozzo [35] reported fumarolic activity records since the pre-Columbian age as the first evidence of volcanic activity. The eruptive history of Popocatépetl is characterized by Strombolian and Vulcanian activity with pumice and ash fall episodes, ballistic emissions, and lahar formations, as well as dome building cycles. The last eruption occurred in the early 1900s. On 21 December 1994, Popocatépetl renewed its activity with phreatic ash emissions after a period of quiescence that lasted about 6 decades. Active dome destruction events followed by open vent periods characterized the active phase of Popocatépetl. Since 1996, an increase in magmatic activity has been recorded, mainly due to the continuous formation and destruction of lava domes [32]. Popocatepetl’s activity currently includes constant passive degassing, low–medium intensity exhalations, moderate explosions, intermittent dome formation and destruction, and the release of toxic gases and ash into the atmosphere.

The petrological and plume composition of Popocatépetl have been described in several published works [33,36]. Witter et al. [37] reported a mixture composition from basaltic/andesitic (current manifestation) to dacitic, with products (phenocrysts and microphenocrysts) that mainly contain the minerals olivine, clinopyroxene, orthopyroxene, plagioclase, and hornblende. The composition of emitted gases has been principally studied using ultraviolet [38] and infrared [39] instruments (i.e., remote sensing techniques). Popocatépetl emits large quantities of sulfur dioxide (SO_2_) and carbon dioxide (CO_2_), and in smaller quantities halogens and gaseous elemental mercury [40,41]. The chemical composition of water leachates of ashes produced in the current eruption period has been reported in several studies [42,43,44].

## 3. Materials and Methods

### 3.1. Samples of Volcanic Ash

Ashfall samples (n = 5, M1 to M5) from Popocatépetl volcano (Lat 19.02222°, Long −98.62778°; elevation 5454 m a.s.l.) were collected during discrete explosive events. Popocatépetl is an andesitic, subduction zone stratovolcano located close to two highly populated cities, Mexico City (~60 km, N-NW direction) and Puebla (~45 km, E direction) with approximately 20 and 7 million inhabitants, respectively. Due to the prevailing winds, the city of Puebla is subject to continuous ashfalls from the volcano, especially during strong explosions. In addition to the megacities, several small communities (around 150,000 total residents) are located in the immediate vicinity of Popocatépetl [38].

About 200 g of tephra samples were collected, put into polyethylene bags, and stored at the National Autonomous University of Mexico, Institute of Geophysics, ICP-MS labs in Mexico City, Mexico. In the laboratory, the samples were placed in a well-ventilated room and subsequently dried at a temperature of 25 °C for 24 h. Before the analyses, samples were mechanically sieved at 100 μm to separate the coarse particles. The tephra samples represent a mixture of extremely fine ash and lapilli.

### 3.2. SEM-EDS Analysis

Morphological and elemental analysis of solid particles was performed on unpolished dry volcanic ash samples mounted on a double-sided carbon tape and sputter coated with a thin carbon layer to improve conductivity. The analyses were carried out in high vacuum mode (~10^−6^ Torr) using a desktop SEM model Thermo Fisher Scientific Phenom Pro X (Waltham, MA, USA), equipped with an EDS for semi-quantitative chemical elemental analysis. The images were recorded during the SEM operation at an accelerating voltage of 15 kV in backscattered electron (BSE) mode, beam current of 0.5 nA, and a working distance of 15 mm. The SEM is a very versatile instrument that allows observation (3D appearance of images) and surface characterization (crystalline structure and orientation) of materials by generating a beam of electrons that impact the sample.

### 3.3. Particle Size Analysis

Particle size distribution analysis was performed by selecting representative images from SEM and using the ParticleMetric Software (PMS). The PMS works in a size range from <100 nm to 0.1 mm and can examine more than 1000 particles per minute. The parameters analyzed were the shape, size distribution, circle equivalent diameter, and circularity (C). Before the particle count analytical procedure, the ash sample was placed in a polyethylene box inside a resuspension chamber with the SEM sampling holder positioned in the center. To reproduce external conditions, i.e., a suspension of a breathable NPs fraction, compressed air was applied inside the chamber, simulating dry deposition by gravity. Experimental details can be found in Meza-Figueroa et al. [45]. For quality control purposes, the procedure was conducted in triplicate.

The hydrodynamic diameter of ash particles in GS and ALF was analyzed using DLS (also called Quasi-Elastic Light Scattering), which is a valuable technique for measuring particle size in the submicron (<1 μm) range. DLS measures the velocity of particle Brownian motion in a fluid and relates this measure to size. The technique is sensitive to the solvent (e.g., distilled water or SLF) around the particles. Particle size is estimated as hydrodynamic diameter because Brownian motion decreases as particle size increases; the measured diameter in DLS refers to the diffusion of the particles in the fluid. The technique can identify particle size distributions from 0.6 nm to 6 μm. DLS measures the temporal fluctuations of the scattered light, from which the translational diffusion coefficient (*D*) is determined and related to the particle size by the Stokes–Einstein equation:

(1) $$d\left(h\right)=\frac{k\cdot T}{3\pi \cdot \eta \cdot D}$$

where *d*(*h*) is the hydrodynamic diameter, *k* is the Boltzmann constant (1.38 × 10^−23^ J/K), *T* is the absolute temperature, and *η* is the viscosity of the dispersing medium. The coefficient *D* also depends on the surface structure, the medium’s ionic strength and the sphericity of particles. If the shape of a particle changes, affecting the diffusion speed, then the hydrodynamic size will also change. The *d*(*h*) of particles in the SLF solution was estimated using a Zetasizer nano ZS instrument (Malvern Instruments Ltd., Malvern, UK).

The GS and ALF solutions (Table S1) were prepared according to the procedure and reagents described by Colombo et al. [46], Kastury et al. [47], Meza-Figueroa et al. [45], and Schiavo et al. [48]. In this study, 0.01 g of ash sample was mixed with 10 mL of each SLF (separate vials), using a solid:liquid (S/L) ratio of 1:1000. The prepared solutions were incubated for 24 h at 37 °C. After the incubation period, the extracts were immediately measured using the DLS technique (0 h).

We analyzed the particle size distribution via DLS in three ash samples (M1, M4, and M5) at different time points (0 to 24 h) to evaluate the agglomeration of submicron particles to NPs in GS and ALF solutions. The measurements were carried out under controlled conditions (external influences complicate the analysis, especially for highly heterogeneous samples), in triplicate, and with a backscatter angle of 173°. As a final result, the technique determines a correlation function between Brownian motion and scattered light intensity as a function of time. Particle size is obtained by applying an algorithm that estimates the width of the distribution expressed as a polydispersity index (PI). The PI is a measure of the heterogeneity of a sample based on the size distribution of particles. Generally, samples with values of PI < 0.05 are considered monodisperse, while samples with values >0.7 are characterized as polydisperse [49].

## 4. Results and Discussion

### 4.1. Morphological and Chemical Analysis

**Figure 2:**
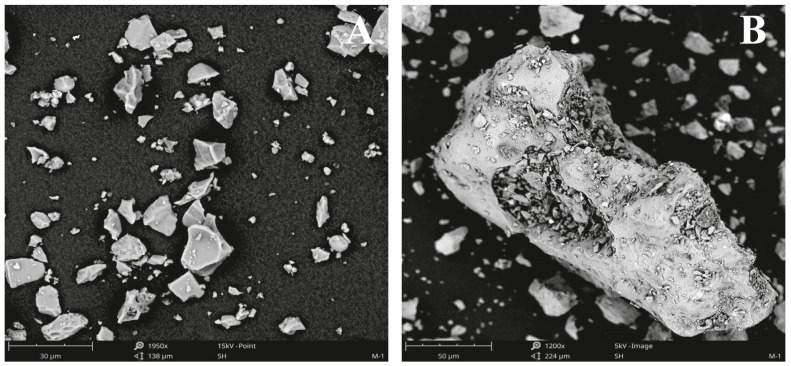
(**A**) General view of particles in ash sample from SEM. Image contains particles with different sphericity, shape, size, and crystallinity. (**B**) Typical particle types and shapes observed in the studied ashes.

An SEM micrograph image of a representative ash sample from Popocatépetl volcano is shown in Figure 2A. The particle morphology of ash samples showed a heterogeneous distribution with various sizes (micro- to nano-sized), irregular and angular shapes, and aggregation. The heterogeneity is due to the internal mechanisms of the volcano, including its degree of fragmentation, in turn caused by the composition of the magma. These kinds of particles are generally associated with natural and volcanic emissions. On the contrary, anthropogenic particles are characterized by more spherical shapes due to combustion processes. Figure 2B shows an SEM image of vesicular particles with a rough surface, sharp edges, and particles less than 1 μm agglomerated with each other. Previous works reported an extremely irregular shape in volcanic ash particles, which reflects fragmentation mechanisms, transport, and environmental complexity [50,51]. Moreover, Diaz-Vecino et al. [52] described the close relationship between agglomeration, sedimentation, and the aerodynamic properties of aggregates. The observed particle size and shape of samples could result from the presence of different mineralogical phases.

**Figure 3:**
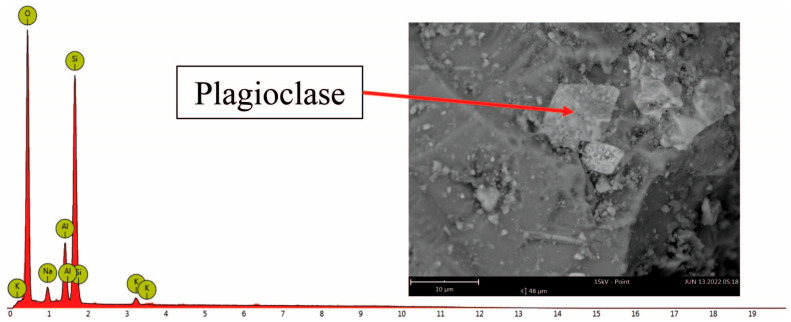
SEM image of Al- and Si-rich particles with EDS spectra.

In this study we detected several mineralogical phases, such as olivine, magnetite, ilmenite, pyroxene, plagioclase, and iron (Fe)—titanium (Ti) oxides, recognized by SEM elemental analysis. This confirms the research carried out by Witter et al. [37], which reported the same minerals in a petrological investigation of magma ejected during the 1997–1998 eruption with a high percentage of Fe, Ti, and aluminosilicate (typical of basaltic/dacitic magma). For instance, Figure S1 shows Fe-Ti oxide, olivine, and pyroxene minerals with irregular shapes, and different textures and morphology. Other crystalline phases, such as plagioclase, were recognized during the elemental analysis (EDS) of bulk samples (Figure 3). The semi-quantitative geochemical composition revealed the presence of many elements, such as Fe, Ti, Silicon (Si), aluminum (Al), magnesium (Mg), sodium (Na), oxygen (O), potassium (K), and calcium (Ca). More elements, such as arsenic (As), barium (Ba), bromine (Br), copper (Cu), sulfur (S), and strontium (Sr), were recognized by the EDS analysis, although they were considered less frequent in the studied ash samples (Table 1). Considering the EDS’s weight (wt%) results, elements such as Fe, Ti, Si, Al, O, and Ca were the most abundant, exceeding 50%. On the other hand, less than 10% by weight was recorded for Mg, Na, and K in ash samples. Generally, a discrete content of Al and Si with variable concentrations of Fe, Mg, K, and Ca is characteristic of aluminosilicates.

### 4.2. Particle Size Distribution

**Figure 4:**
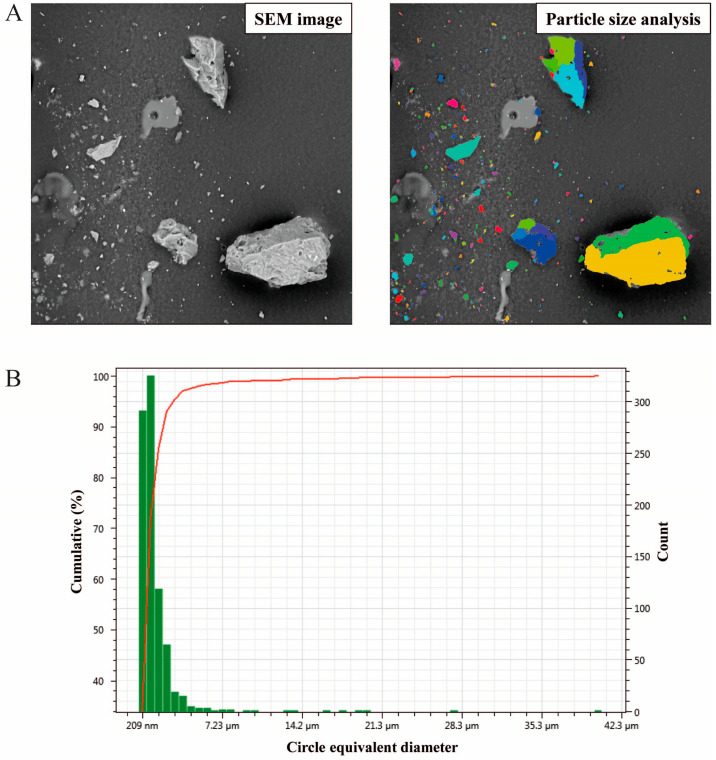
(**A**) Example of an SEM image (**left**) and particle count from ParticleMetric software (PMS) (**right**). (**B**) Barplot of PMS results in ash sample M3.

The particle size distributions obtained by PMS-SEM analysis for five studied ash samples (M1 to M5) are presented in Table 2. Furthermore, Figure 4A displays how the PMS software detects the different sizes of particles starting from a raw SEM image.

The distribution of ash particle size was classified according to the following descending order fractions: <20 μm (PM_20_), <10 μm (PM_10_), <5 μm (PM_5_), <2.5 μm (PM_2.5_), and <1 μm (PM_1_). Circularity was found to be relatively low in the ash samples under consideration, with normalized values lower than 0.73. In general, for natural particles, the circularity should be less than 0.8. Only in certain cases, such as anthropogenic particles [53] or natural particles that have been transported over long distances [45] (in media like air or water), can higher sphericity and circularity (i.e., greater roundness) be reached. Particles characterized by low circularity and small size suggest no long-distance transport into the environment. Additionally, particle size (i.e., average equivalent circle) was found to be between 1.61 and 3.89 μm, with an average of 2.71 μm. The lowest equivalent circle was registered in sample M3 (1.61 μm), followed by samples M4 (2.05 μm), M1 (2.37 μm), M5 (3.67 μm), and M2 (3.89 μm). In all the studied ash samples except for M2, abundant PM_1_ particles were detected compared to the other sizes (PM_2.5_, PM_5_, PM_10_, and PM_20_). In particular, sample M3 was the only case in which about 85% of the particles analyzed were concentrated in the PM_1_ and PM_2.5_ fractions (Figure 4B). In samples M1, M3, and M4, the proportion of PM_1_ particles was higher than 50%, with values of 64.70%, 71.79%, and 53.80%, respectively. On the other hand, in samples M2 and M5 the amount of PM_1_ particles was lower than 50%, with values of 7.86% and 33.42%, respectively. Sample M2 was the only case with the majority of the particles, approximately 43.67%, concentrated in the PM_5_ fraction (Figure S2).

The range of these particles (from PM_20_ to PM_1_) was considered because of their ability to penetrate into the lower respiratory tract region. More specifically, particles smaller than 2.5 μm can easily penetrate up to the alveolar macrophage (deep lung area). The PM_1_ particles (submicrometric fraction) are even more dangerous, reaching the bloodstream and crossing the cell wall [54]. The ash particle size distribution in volcanic systems depends on several factors, from magma fragmentation and ascent rate to external factors such as particle collision and environmental interactions. Starting material inside the volcanic conduit is characterized by a dense structure and greater size compared to ejected ashes. Once emitted, the ashes (especially submicron and nano-sized) are subject to aggregation processes [55], which influence the behavior of the particles in the atmosphere and the velocity of falling. Brown et al. [56] described that ashfall with <63 μm size has a greater propensity to aggregate and form larger and lower-density particles. Moreover, Beckett et al. [57] reported a typical diameter higher than 63 μm for aggregated ash particles. Particle aggregation in volcanic ash occurs under specific forces, mainly hydro-bonds (liquid and ice water) and electrostatic forces [57]. Instead, Hotze et al. [58] describe a type of aggregation called homoaggregation that characterizes the relationships between similar particles (e.g., NPs) combined by Brownian diffusion. This process is typical of volcanic ash aggregations, as confirmed by Trejos et al. [23], reporting NP aggregations of approximately 10 nm.

### 4.3. Particle Polydispersity in Simulated Lung Fluid

**Figure 5:**
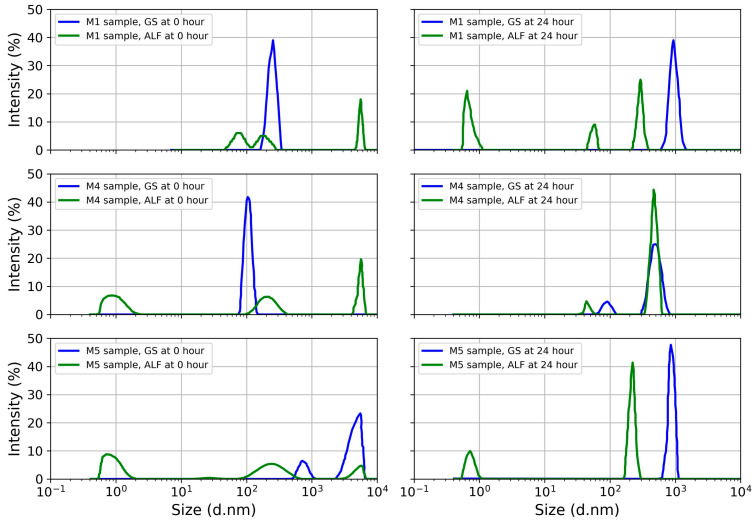
Analysis of the particle size distribution of ashfall samples M1, M4, and M5 by DLS at 0 to 24 h incubation in GS and ALF.

The results of DLS analysis reported in Table 3 indicate an elevated polydispersity index and extremely ultrafine particles in the ash samples. The index of polydispersity values agrees with data previously published by Lädhe et al. [21] for Icelandic volcanoes. The PI increased in the ALF solution with a longer exposure time (24 h). In the polydisperse samples, sedimentation and agglomeration were observed in the ashfall. In the simulated GS medium, after 24 h of incubation of the ashfall, aggregation was observed in all samples, while disaggregation was observed in the ALF medium. Aggregation in the GS medium is evidenced by the increase in average hydrodynamic diameter from 240 nm at the starting time to 963.6 nm after 24 h of exposure (M1). In the case of the ALF solution simulating the interior of the alveolar macrophage, a decrease in the average hydrodynamic diameter of particles (disaggregation) was observed, ranging from 209.4 nm at the initial exposure time to 45.3 nm and even 1 nm at 24 h of exposure (M4). Average hydrodynamic diameter close to 1 nm was detected in samples M1 and M5 during 24 h exposure of the ALF solution (Figure 5). Moreover, as shown in Figure 5, the signal intensity was generally higher in the GS compared to the ALF solution. This variability can be explained by the different pH values between the two simulated solutions—neutral (pH~7) for GS and acid (pH~4.5) for ALF. In particular, the corona protein (CP) effect could explain the rapid aggregation of ash NPs in GS solution. Konduru et al. [59], through in vivo studies on rats, report that the CP effect is more effective in the lung lining fluid. Furthermore, depending on the nature of the NPs (composition, mineralogy, and shape), the absorption of proteins can be highly variable. The trend was similar for all samples studied, regardless of particle size distribution and signal intensity. In addition, more variability was present regarding the hydrodynamic diameter of the ash, considering that NPs were found in all samples.

Previous studies reporting particle size distribution using DLS include those of Lähde et al. [21] and Kendall et al. [60]. However, these works use distilled water as the suspension medium. It should be considered, however, that the particles, once inhaled, encounter biological fluids containing polymers such as fibrinogenic proteins. Published works show in vitro aggregation for submicron silica particles and indicate that this aggregation depends on the type of particle surface [60]. Considering that the DLS methodology is solvent-sensitive, it is essential to use synthetic lung fluids to better understand the behavior of ash particles within the lung.

As reported in the literature, aggregated particles tend to settle [61]. Previous studies indicate that aggregated NPs of TiO_2_ or black carbon can disaggregate into smaller particles and transfer from alveolar spaces to cellular interstitial sites with unknown chronic effects [62,63]. Kreyling et al. [61] showed that the translocation and accumulation of particles in tissues depend on material type and aggregation. The study showed that 80 nm particles aggregated whereas 20 nm particles disaggregated, translocated, and accumulated in tissues. Kendall et al. [60] suggest that particle aggregation is a protective mechanism and that the lung lining fluid modifies the chemistry by affecting the attractive forces on the particle surface to promote the agglomeration mechanism.

The behavior of the ash particles in the ALF solution at 24 h of exposure indicates the disaggregation of particles as small as 1 to 79 nm. The potential particle translocation to tissues represents an uncertain health risk, and it deserves further study. Horie and Tabei [64] report that NPs have the ability to induce primary and secondary oxidative stress through the generation of intercellular reactive oxygen species. Variable particle size distributions and shapes for volcanic ashes have been reported. Ashes from Icelandic volcanoes range from 669 to 940 nm [21], and those from Klyushevskoy volcano in Russia have three ranges, from 45 to 100 nm, 100 to 400 nm, and 400 to 830 nm [65], and show variable aggregation behavior in aqueous media [14]. Our results suggest the relevance of evaluating the particle size distribution of volcanic ash particles in synthetic lung media, since it is unknown whether ashes from other volcanoes show disaggregation in alveolar macrophage solution.

## 5. Conclusions

The present work has been focused on the study of NPs contained in volcanic ash. Advanced microscopic analysis was used to determine the morphology, mineralogy, chemical composition, and particle size distribution of ash samples, and to evaluate the polydispersity of ash NPs in SLFs.

In general, irregular and angular-shaped particles typical of volcanic ash were observed during the SEM analyses. SEM-EDS analysis made it possible to identify major elements such as Si, Al, Fe, Ca, Mg, O, K, Na, and Ti, and potential toxic elements such as As and Cu. The PMS detected submicrometric particles (i.e., PM_1_) in all analyzed samples, with an average equivalent circle diameter of 2.72 μm. In some cases, such as sample M3, the submicrometric particles exceeded 70% of the total amount, with particles that reached an equivalent circle diameter of up to 200 nm. Ash particles in contact with lung synthetic solutions showed high polydispersity and variable hydrodynamic diameter, with values from 0.31 to 1 and from 0.71 to 5560 nm, respectively. Aggregation processes were detected in the GS after 24 h of interaction with volcanic ash. On the other hand, after 24 h of exposure to the ash in the ALF solution, disaggregation processes were identified. This last process made it possible to observe by DLS extremely ultrafine particles, with sizes close to 1 nm, after 24 h of exposure to the ALF solution.

The findings of this work make it clear that future studies on NPs emitted by volcanic eruptions should be investigated in lung fluids, focusing on exposure time, aggregation and disaggregation processes that control the fate and behavior of NPs in the human body.

## Figures and Tables

**Table 1:** Particles’ chemical composition obtained from studied ash samples by SEM-EDS.

Sample	M1	M2	M3	M4	M5
Element					
Al	x	x	x	x	x
As	x				
Ba	x				
Br	x	x			
Ca	x	x	x	x	x
Cu			x	x	
Fe	x	x	x	x	x
K	x	x	x	x	x
Mg	x	x	x	x	x
Na	x	x	x	x	x
O	x	x	x	x	x
S	x		x		
Si	x	x	x	x	x
Sr	x	x		x	x
Ti	x	x	x		x

**Table 2:** Particle size analysis obtained by ParticleMetric software (PMS) of ashfall from Popocatépetl volcano. The values of particulate matter (PM) with different aerodynamic diameters are reported as percentages (%).

Sample	Number of Particles	PM_1_	PM_2.5_	PM_5_	PM_10_	PM_20_	Equivalent Circle (μm)	* Circularity
M1	694	64.7	10.4	17.3	5.9	1.7	2.4	0.67
M2	229	7.8	27.9	43.7	14.4	6.1	3.9	0.72
M3	858	71.8	13.7	12.0	1.4	1.1	1.6	0.43
M4	303	53.8	27.7	13.5	3.9	0.9	2.1	0.53
M5	377	33.4	20.2	29.2	11.7	5.6	3.7	0.51

* Values close to 1 represent particles with the most spherical shape.

**Table 3:** Polydispersity index and hydrodynamic diameter, analyzed at different time points (0 to 24 h), of three ash samples in GS and ALF solution.

	GS		ALF	
**Time**	0 h	24 h	0 h	24 h
**M1**				
Polydispersity index	0.77	0.65	1	1
Hydrodynamic diameter (d.nm)	240	963.6	79, 184.9, and 5560	0.65, 55.3, and 291.9
**M4**				
Polydispersity index	-	0.76	0.95	1
Hydrodynamic diameter (d.nm)	109.7	89.7 and 498.1	0.91, 209.4 and 5367	45.3 and 468.7
**M5**				
Polydispersity index	0.35	0.89	0.31	1
Hydrodynamic diameter (d.nm)	749.3 and 4516	864.9	0.72, 251.7, and 4857	0.71 and 216.1

## Data Availability

The data presented in this study are available on request from the corresponding author.

## Supplementary Materials

The following supporting information can be downloaded at https://www.mdpi.com/article/10.3390/toxics11070624/s1, Figure S1: Mineralogical phases recognized in ash samples. A: Fe-Ti oxide, B: Olivine, C: Pyroxene. Figure S2: Particle size distribution by ParticleMetric software (SEM) of M1, M2, M4, and M5 ash samples. Table S1: Chemical composition of Gamble solution (GS) and Artificial lysosomal fluid (ALF) solution used for in vitro lung bioaccessibility.
