# Supplementary material for: Characterization and Polydispersity of Volcanic Ash Nanoparticles in Synthetic Lung Fluid
Source: Toxics. 2023 Jul 19;11(7):624. doi: 10.3390/toxics11070624 (PMC10383943; doi:10.3390/toxics11070624)
Supplement: Supplementary file 1 [file toxics-11-00624-s001.zip › toxics-2485078-supplementary.pdf]

Characterization and Polydispersity of Volcanic Ash Nanoparticles in Synthetic Lung Fluid

Benedetto Schiavo[1*], Ofelia Morton-Bermea[1], Diana Meza-Figueroa[2], Mónica Acosta-Elías[3], Belem González-Grijalva[2], Maria Aurora Armienta-Hernández[1], Claudio Inguaggiato[4], Daisy Valera-Fernández[1]

Supplementary meterial

Figure S1: Mineralogical phases recognized in ash samples. **A:** Fe-Ti oxide, **B:** Olivine, **C:** Pyroxene.

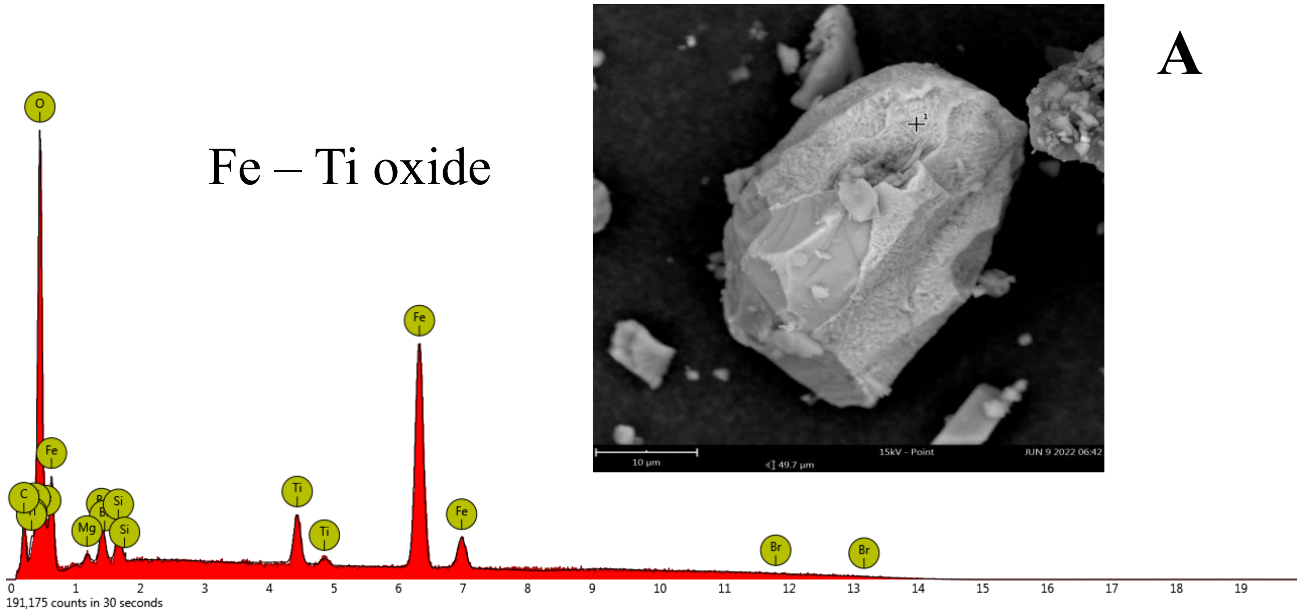

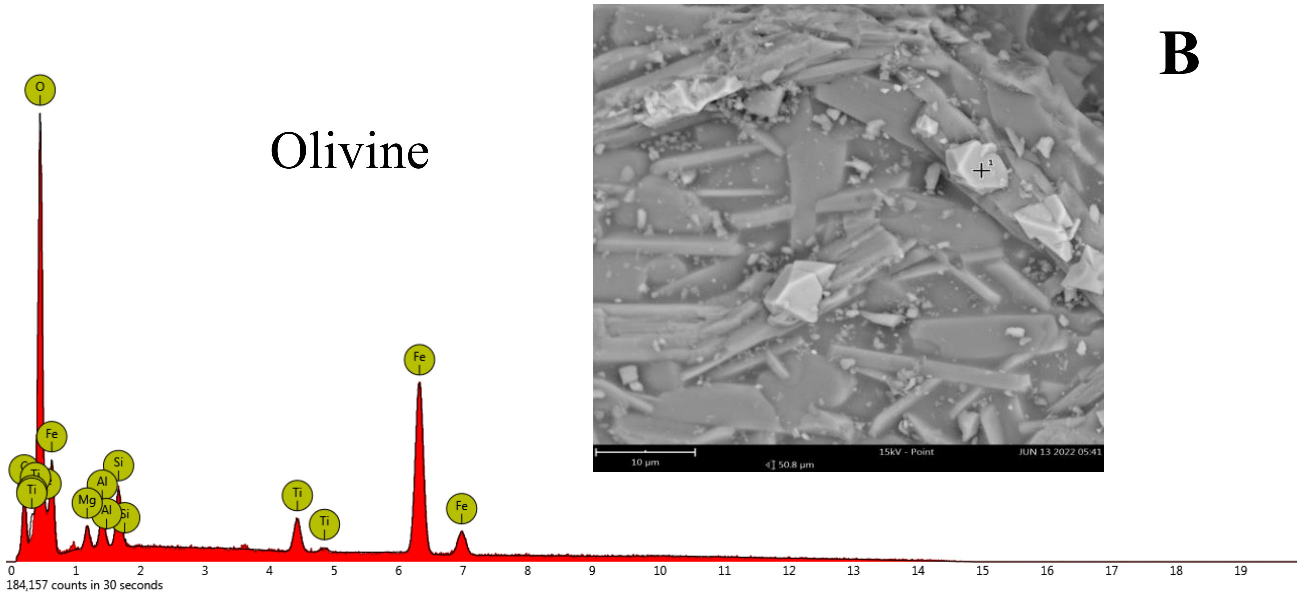

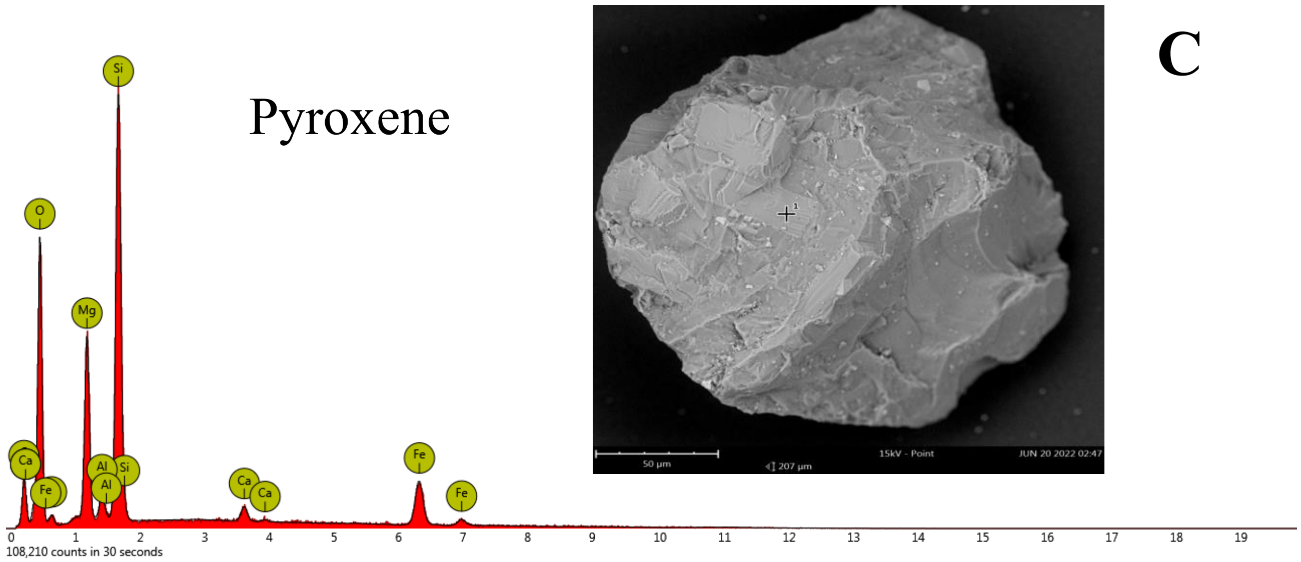

Figure S2: Particle size distribution by ParticleMetric software (SEM) of M1, M2, M4, and M5 ash samples.

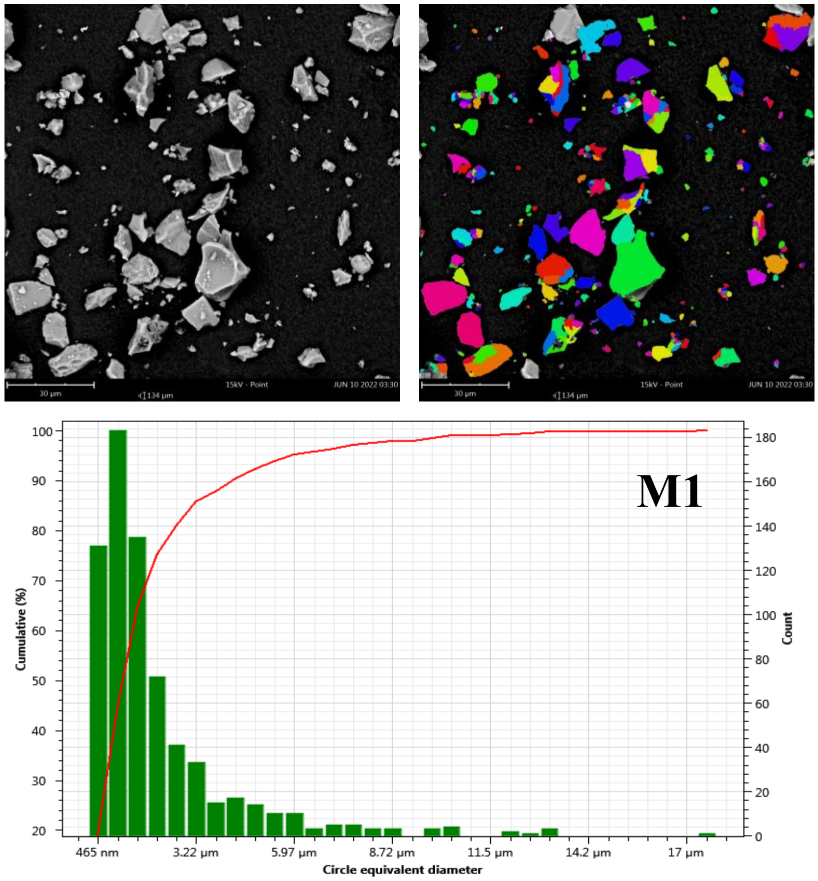

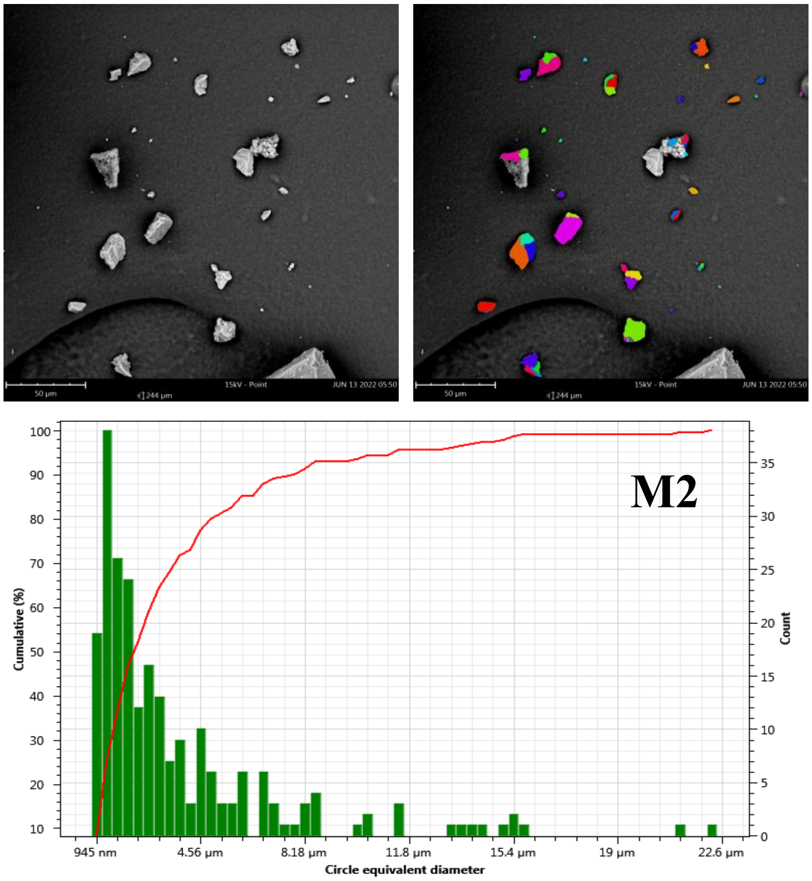
M2
50 µm
15kV - Point
JUN 13 2022 05:50
Cumulative (%)
Count
945 nm
4.56 µm
8.18 µm
11.8 µm
15.4 µm
19 µm
22.6 µm
Circle equivalent diameter

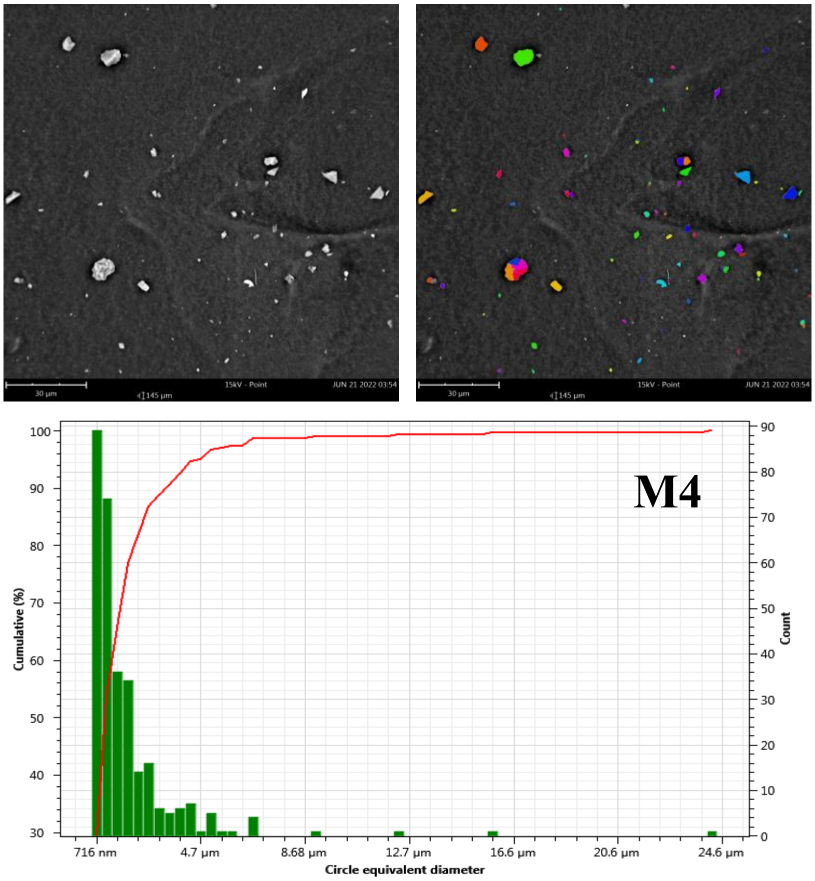
M4
30 µm
15kV - Point
JUN 21 2022 03:54
Cumulative (%)
Count
716 nm
4.7 µm
8.68 µm
12.7 µm
16.6 µm
20.6 µm
24.6 µm
Circle equivalent diameter

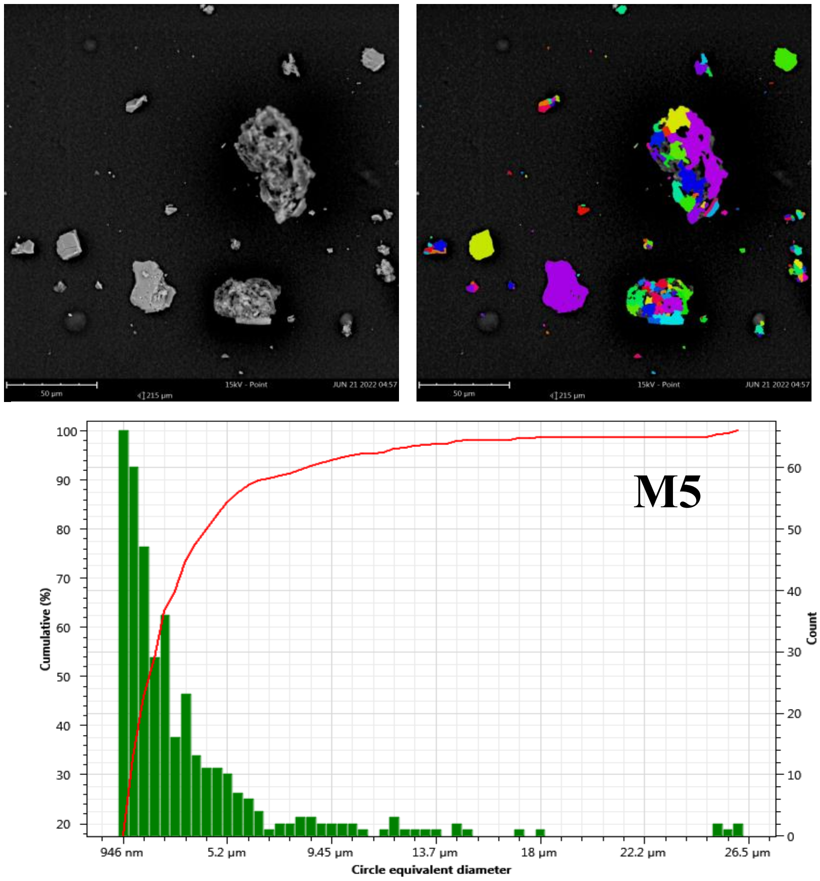
50 μm
215 μm
15kV - Point
JUN 21 2022 04:57
50 μm
215 μm
15kV - Point
JUN 21 2022 04:57
M5
Cumulative (%)
Count
100
90
80
70
60
50
40
30
20
60
50
40
30
20
10
0
946 nm
5.2 μm
9.45 μm
13.7 μm
18 μm
22.2 μm
26.5 μm
Circle equivalent diameter

Table S1: Chemical composition of Gamble solution (GS) and Artificial lysosomal fluid (ALF) solution used for in vitro lung bioaccessibility.

Reagent required for 1 L of solution (g)	Formula	GS (pH ~ 7.4)	ALF (pH ~ 4.5)
Magnesium chloride hexahydrate	$MgCl_2{\cdot}6H_2O$	0.10	0.05
Sodium chloride	$NaCl$	6.02	3.21
Potassium chloride	KCl	0.30	-
Disodium hydrogen phosphate	Na_2HPO_4	0.13	0.07
Sodium sulphate	Na_2SO_4	0.06	0.04
Calcium chloride dihydrate	$CaCl_2{\cdot}2H_2O$	0.37	0.13
Sodium acetate	$C_2H_3O_2Na$	0.57	-
Sodium hydrogen carbonate	$NaHCO_3$	2.6	-
Sodium citrate dihydrate	$C_6H_5Na_3O_7{\cdot}2H_2O$	0.10	0.08
Sodium hydroxide	$NaOH$	-	6
Citric acid	$C_6H_8O_7$	-	20.8
Glycine	H_2NCH_2COOH	-	0.06
Sodium tartrate dihydrate	$C_4H_4O_6Na_2{\cdot}2H_2O$	-	0.09
Sodium lactate	$C_2H_5NaO_3$	-	0.09
Sodium pyruvate	$C_3H_3O_3Na$	-	0.09
